# Indoor Pedestrian Navigation Using Foot-Mounted IMU and Portable Ultrasound Range Sensors

**DOI:** 10.3390/s110807606

**Published:** 2011-08-02

**Authors:** Gabriel Girard, Stéphane Côté, Sisi Zlatanova, Yannick Barette, Johanne St-Pierre, Peter van Oosterom

**Affiliations:** 1 Department of Computer Science, Université de Sherbrooke, 2500 boulevard de l’Université, Sherbrooke J1K 2R1, QC, Canada; 2 Applied Research Group, Bentley Systems, 3645 boulevard Sainte-Anne, Québec G1E 3L1, QC, Canada; E-Mails: stephane.cote@bentley.com (S.C.); johanne.st-pierre@bentley.com (J.S.-P.); 3 Section GIS Technology, OTB, Delft University of Technology, Jaffalaan 9, Delft 2628 BX, The Netherlands; E-Mails: s.zlatanova@tudelft.nl (S.Z.); p.j.m.vanoosterom@tudelft.nl (P.O.); 4 Department of Electrical Engineering, Université Laval, 2325 rue de l’Université, Québec G1V 0A6, QC, Canada; E-Mail: yannick.barrette.1@ulaval.ca (Y.B.)

**Keywords:** indoor localization, inertial tracking, model-based navigation, ultrasound range sensors

## Abstract

Many solutions have been proposed for indoor pedestrian navigation. Some rely on pre-installed sensor networks, which offer good accuracy but are limited to areas that have been prepared for that purpose, thus requiring an expensive and possibly time-consuming process. Such methods are therefore inappropriate for navigation in emergency situations since the power supply may be disturbed. Other types of solutions track the user without requiring a prepared environment. However, they may have low accuracy. Offline tracking has been proposed to increase accuracy, however this prevents users from knowing their position in real time. This paper describes a real time indoor navigation system that does not require prepared building environments and provides tracking accuracy superior to previously described tracking methods. The system uses a combination of four techniques: foot-mounted IMU (Inertial Motion Unit), ultrasonic ranging, particle filtering and model-based navigation. The very purpose of the project is to combine these four well-known techniques in a novel way to provide better indoor tracking results for pedestrians.

## 1. Introduction

Measuring in real time the exact position of a user indoor is essential to many applications like augmented reality and indoor navigation for emergency situations. Some systems that have been proposed for that purpose are based on prepared environments: they rely on pre-installed devices that can be detected by sensors carried by the user (e.g., RFID, ultrasound beacon, *etc.*). Such systems can provide good positional accuracy. However, since they require preparation of the tracking area, they apply only to a subset of potential applications and they may stop functioning in emergency situations. Other solutions that can be used in unprepared environments use portable sensors such as accelerometers, gyroscope and magnetometers, which may be supported by vision systems [1,2]. Such systems can be applied in any environment (indoor and outdoor) and are therefore very suitable for emergency management applications. However the accuracy of the positioning might be lower than the one obtained with systems designed to work in prepared environments.

State-of-the-art methods for indoor localisation combine portable motion sensors to pre-installed devices [3–8]. Renaudin *et al.* [4] proposed a hybrid solution that combines dead reckoning and RFID beacons: waypoints are dynamically installed to decrease the bias and drift associated with dead reckoning methods. The first leading user deploys an RFID beacon at a defined location and the following users take advantage of the known location of the beacon to estimate their position. This solution is more flexible than a positioning system based on existing sensor networks since installation of the beacon can be done quickly, and it improves dead reckoning positioning. However, the position of the RFID beacons must be known, and they still have to be installed prior to navigation. For complex buildings, we may assume that the number of required beacons would be high.

The motivations for this work are applications similar to navigation of emergency responders that require good indoor localization (1–2 m) without requiring a prepared building environment. Furthermore, the sensor equipment should be light, inexpensive, and effective in areas of reduced visibility. In our approach we assume that a model of the building (floor plans) is available and all the computation for estimating user location is performed on a portable computer (tablet, smartphone) carried by the user. The main problem of a fully portable solution is accuracy. We hypothesized that the accuracy of existing systems could be increased using an additional sensor often used in robotics: a portable ultrasound range sensor. Portable ultrasound sensors can, like IMUs, be very small in size and be installed on the user in such a way that they do not disturb his normal movements. Our proof-of-concept developments have clearly shown an significant accuracy improvement.

This paper is organized as follows. The next section provides a background about the technologies used in our approach. We then describe the proposed tracking method in Section 3. We discuss the results of the tests in Section 4. The final sections provide a conclusion and an outline of proposed future research work.

## 2. Related Work

The use of various technologies has been proposed in the literature to solve localization and tracking problems. This work is based on the use of four well-known technologies: foot-mounted IMU, ultrasonic range measurements, particle filtering and model-based navigation. These will be briefly explained below.

### 2.1. Foot-Mounted IMU

An IMU is fixed on the foot of the user. Its acceleration and orientation measurements are used to detect the user’s steps and to estimate their length and orientation. The method is based on the use of a technique called “zero velocity updates”: at each step, the foot touches the ground, at which time we know that its speed with respect to the ground is zero, and therefore the integration process can be re-initialized, resetting any drift. The method has been used in several investigations [7,9–15].

### 2.2. Ultrasonic Range Measurements

Ultrasound range sensors have not been used for tracking humans but they have been used to control robots in an indoor environment [16–19]. They have been employed for obstacle avoidance, position estimation as well as to provide a basis for the development of a wall-following device by Bemporad *et al.* [16], for curb sensors in vehicles, for measuring levels of liquids in tanks, *etc.* Ultrasound range sensors consist of a piezoelectric crystal that is periodically excited to produce cyclic compression waves of a frequency normally exceeding 40 kHz. The waves then travel through air, hit objects, and bounce back. By measuring the round trip time between emission of the wave and its reception, the sensor can calculate the distance traveled by the wave, knowing that the waves travel at the speed of sound which is constant for a given set of air properties (e.g., density, temperature, *etc*.) [20].

### 2.3. Particle Filtering

In pedestrian navigation, a particle filter could be described as a probabilistic distribution of points based on the measurement. Particles together form a cloud which represents the belief of the user position over time. Each particle is characterized by:

- A weight *w*, based on probability (its influence in the cloud).
- A position *p*, absolute or relative, within the cloud.

As the user moves, the resulting position area is represented by a distribution of particles on a map. The size of the distribution is related to the error of the motion measurements. An algorithm is then applied to find the most probable user position based on the distribution of particles and their weight. The particle filter method has been used in several investigations [9,11,14,17–19,21].

### 2.4. Model-Based Navigation

A model-based navigation system uses a vector model of the area of interest to improve the estimate of the user’s position. It combines the use of model features (such as walls, open areas or obstacles), information from the sensors (such as speed and direction) and information from the user (such as human behavior) to find the most plausible user’s position in the model. Model-based navigation has been used in [9,10,14,17,21,22].

### 2.5. Combination of the Selected Technologies

Our approach uses a combination of particle filters, vector model and ultrasound range sensors measurements to improve the localization obtained by the foot-mounted IMU. The combined use of portable ultrasound range sensors with those technologies for human motion tracking detection is the novel aspect and main contribution of this work.

The concept of particle filter combined with model-based navigation is an excellent approach for representing the approximate position of a user. It allows individual treatment of particles with respect to other conditions and constraints. However, in the algorithm proposed by Krach *et al*. [9], particles have to move all the way to a physically impossible position, e.g., through a wall, before being eliminated. The uncertainty of the user’s position is directly correlated to the size of room in which he is located. This may result in a large cloud that often fills the area of the room correlated. Since the estimated position of the user is based on the location (and the weight) of those particles, the positional accuracy suffers from the size of the cloud. A method is needed that would let the system eliminate particles before they even reach the walls.

To fulfill that need, we propose the use of portable ultrasound range sensors. Ultrasound sensors have already been used to assist human position tracking methods. Fischer *et al*. [7] deployed ultrasound beacons, the sound from which was detected by sensors worn by the user. Measurements were used to lower estimation errors of the user position. Similar to the dead reckoning with RFID beacons [4], this approach relies on existence of two teams, *i.e*., the first one placing the ultrasonic beacons and the second one making use of it. The ultrasound sensors we use are small, low powered, and can therefore easily be worn by the user. We proposed to install them in such a way to measure the distances between the user and adjacent walls. By comparing those distance measurements with the distance between particles in the cloud and their surrounding walls in the model, we could eliminate particles when their position with respect to the walls disagrees with the measured ranges. This has the potential of dramatically increasing the precision of model-based and dead-reckoning user position estimation, as particles will be eliminated before they even reach walls, therefore decreasing the size of the cloud.

### 2.6. Indoor Ultrasound Ranging Issues

In this work, we used two Devantech SRF02 Ultrasound Range Finders, attached in a back-to-back configuration, to measure the distance to the walls adjacent to the user. Those sensors are characterized by a minimum range of 15 cm, a maximum range of 600 cm, an average range error of 3 to 4 cm and a ranging cycle of 70 ms.

Ultrasonic ranging is characterized by several issues that must be taken into account in the interpretation of the ranging data and affect the way the sensors can be used. Some of them are described below.

#### 2.6.1. Wave Reflection

**Figure 1.**
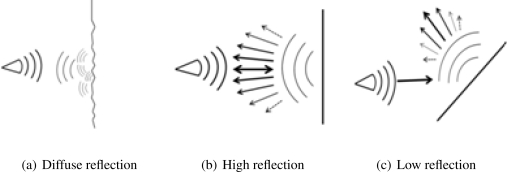
(**a**) Diffusion of signal caused by textured surface; (**b**) The incidence angle is small, so maximum intensity is returned to the sensor; (**c**) At higher incidence angle values, the returned signal is much weaker.

Ultrasonic waves reflect on most solid objects. However, reflection is generally attenuated, depending on various parameters, including: acoustic impedance of the air and of the object [20], diffusion caused by surface texture, angle of incidence of the wave with respect to the surface of the object (see Figure 1).

Our tests with the SRF02 have shown that ultrasonic waves rebound well on all surfaces that we tested: hard floor and wall, carpet, curtains, glass, *etc.*, as we were able to measure range on all those surfaces. It turned out that the most influent parameter was the angle of incidence of the wave, and best results were naturally obtained with near-zero incidence angle values.

#### 2.6.2. Angular Response

**Figure 2.**
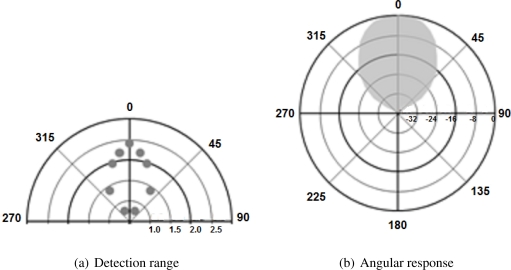
(**a**) Maximum detected ranges (in meters) to a 55 mm plastic pipes for selected angles, as described in [23]; (**b**) Approximate angular response in dB of the SRF02 sensor used in the proposed system at half range, as described in [23].

Ultrasound sensors have a circular opening that lets the waves out in a conical shape with a certain aperture value. Maximum wave power is obtained directly along the sensor’s axis, as is maximum range. Wave power decreases towards the edges of the cone, so maximum range is lower in that zone. This is illustrated in Figure 2(a), where the maximum distance at which a 55 mm diameter pipe can be detected by the Devantech sensor is shown for various angles. The angular response of the SRF02 sensor in dB is illustrated in Figure 2(b).

#### 2.6.3. Wave Attenuation

**Figure 3.**
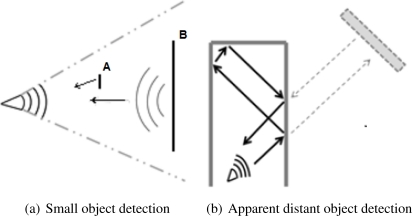
(**a**) The small object A is not detected despite being the nearest, because its surface is too small; (**b**) Secondary reflections hitting obstacle and being detected. The result is an apparent distant object.

The total acoustic power returned to the sensor is proportional to the perceived surface from the sensor (cross-section) and inversely proportional to the square of the distance to the obstacle [20]. Consequently, the sensor may fail to detect objects that are either too small or too far away. Similarly, the sensor may detect a large distant object but not a small, closer one (see Figure 3(a)).

#### 2.6.4. Reverberation

The main consequence of the limited angular response of the sensor is that with increasing values of the wave incident angle, the returning wave may become undetectable. At such point, two things may happen:

- The reflected wave continues to travel until it fades after maximum range yielding no ranging data.
- The wave bounces several times, for instance between two parallel walls (See Figure 3(b)). That situation, common in indoor environments, leads to false measurements.

#### 2.6.5. Ranging Sequence

Ultrasound range sensors run in cycles: sending the wave, starting a timer, waiting for echo to be received, then stopping the timer once received. The maximum range of the sensor is determined by the power of the wave, but also by the timer. The SRF02 timer threshold is set to 70 ms. If the sensor has not received an echo 70 ms after sending the wave, it returns an “out of range” message and it is then ready to start a new send-receive cycle.

Since the sensor is controlled programmatically by way of a virtual COM port over USB, the communication system is characterized by a latency of about 1 to 4 ms, which does not allow us to drive the triggering of the wave burst (*i.e.*, the ping) manually nor to handle the timing. In a back-to-back sensors configuration such as the one proposed, a sensor may receive the wave from the other sensor because of reverberation. However, since we have no control over timing, it is impossible to directly discriminate from which sensor the wave came if the 70 ms limit is not elapsed. We have identified several potential solutions to this problem but they all involve direct control and precise timing of the transducer that can only be provided via embedded hardware.

Consequently, the best course of action in a USB-controlled back-to-back sensor configuration is to get ranging measurements sequentially, waiting from the first sensor to receive the ranging data before firing the second sensor. That actually decreases spatial resolution along the walls, but it nearly guarantees no false readings caused by cross-reverberation.

#### 2.6.6. Detection of Openings

**Figure 4.**
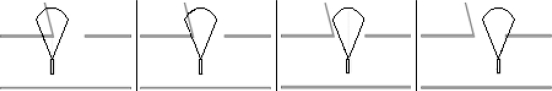
As a sensor moves along a corridor next to an open door, it detects the frame until no part of it is inside the area covered by the sensor.

**Figure 5.**
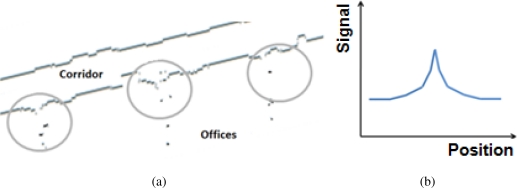
(**a**) Range data collected during a survey test in a corridor. Door frames are detected on one side, along with some posts; (**b**) Representation of the typical signature of a door opening.

Since a sensor detects the nearest object in its beam and because our sensor’s aperture is rather large, openings such as door frames give only a modest signature on ranging data. This is illustrated in Figure 4. We ran an experiment aimed at verifying whether we could easily detect opened doors using the sensors. A user was walking in a corridor with the sensors in a back-to-back configuration. A sample of the data is shown in Figure 5(a), where 3 door frames can be identified. A representation of the typical signature provided by such openings is shown in Figure 5(b).

In spite of those limitations, the use of ultrasound range devices for indoor localization has a lot of potential. Ultrasound can travel through smoke, is not sensitive to electromagnetic fields, and cannot be heard by the human ear, all of which make it an excellent choice for emergency situations. Ultrasound range devices provide low resolution ranging data but can be used advantageously to complement dead reckoning and model based navigation.

## 3. Implementation and Tests

The proposed tracking system is based on a combination of presented technologies. The vector model used was generated from the floor plan of the building in which the tests were completed. To speed up the computation we sub-divided the model into virtual square tiles of 2 *m* × 2 *m* (tile size was chosen after several performance tests in the tracking area). This allows the system to interact with only a sub-set of vector data to calculate collisions, which enhances performance.

### 3.1. Parameters

**Figure 6.**
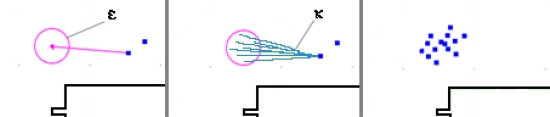
Step sensing error procedure. The system measures a motion vector and sets a plausible error *∈* around it (**left**). The system generates *κ* vectors within the error (**center**). Each of the generated vectors is applied to every particle, resulting in a new, larger cloud (**right**). Particles of the previous step are removed.

**Figure 7.**
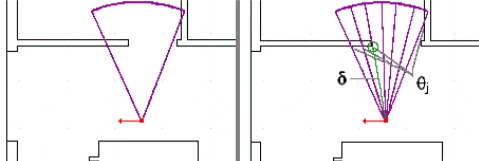
The rays sampling approach. The user position is marked by the square, his walking direction indicated by the arrow. The cone represents the area covered by a range sensing device (**left**). The area is sampled into rays. The circle indicates a plausible obstacle reflection, *δ* is the distance calculated and *θ_j_* indicates ray-obstacle collisions angle (**right**).

The developed algorithms are dependant on five important parameters:

- *ν*: The maximum number of particles in the cloud. If this number is too low, the accuracy of the system decreases; holes appear in the cloud when it becomes too wide. To ensure a relatively uniform distribution of particles within the cloud, *ν* should be given a large enough value. However, *ν* should be set sufficiently small to ensure real-time operation of the system.
- *ε*: The estimated size of the error (in centimeters) on the step measurement, which is the maximum error the system can handle on a single step measurement. We assume the step measurements are unreliable, and *ε* is the confidence level in the accuracy of the IMU measurements. The larger the value of *ε*, the larger the cloud will become. Larger values will result in a decrease of the overall precision of the tracking system. On the other hand, if that number is too small, the system could underestimate the error on measurements, thus the IMU could provide erroneous measurements that could not be handled by the system (see Figure 6).
- *κ*: The number of re-sample vectors generated for each particle. For each step, the system will randomly generate, using a uniform distribution, a set of *κ* vectors around a radius of *ε* of the motion vector measured by the IMU. Those vectors will be applied to each particle in the cloud to generate new particles that represent potential movement of the user. *κ* is strongly related to *ε*; it must be set sufficiently high to provide a relatively uniform distribution of vectors to properly represent user motion within the area covered by *ε*. However, the higher the value of *κ*, the more time will be required to calculate the position of particles.
- *τ*: The minimum distance (in centimeters) between particles in the cloud. This parameter will help obtaining a better distribution of particles within the cloud. When the distance between two particles is below *τ*, those particles are merged. This keeps the number of particles down to a manageable number. Two particles at the same position increase the computation requirements while having little influence on accuracy. The two particles move in the same way, generate the same new particles, and eventually will be removed at the same time. The only thing that matters is their weight, since weight will influence the user’s final estimated position. Therefore, the particle with the lowest weight is removed and its weight is added to the other particle.
- λ: The number of rays used to split the aperture angle of the ultrasound to calculate the distance to wall. This method is detailed below and illustrated in Figure 7. Increasing the number of rays improves precision but also computational requirements. If the value is too small, some elements of the model could be missed because the sampling is insufficient.

These parameters must be well chosen to ensure real-time calculation and good system accuracy.

### 3.2. Assumptions

The tracking process can be divided in 8 steps:

1. Starting position is manually initialized by the user, on the map of the building. A single particle representing the current user position is added, creating the cloud. If the system gets lost, or needs re-initialization, this step must be re-done.
2. The system waits for a motion event. User motion is tracked by measuring the length and orientation of each step with the foot-mounted IMU.
3. From the step measurements and the associated error *ε*, the system randomly generates a set of *κ* vectors that are within the margin of error (*i.e.,* a set of plausible motion vectors) (see Figure 6).
4. Each of the vectors generated in the previous steps are applied to every particle of the cloud to create new particles (see Figure 6). The model limits displacement of particles to physically plausible positions, eliminating those that are unrealistic. Particles of the previous step are also removed.
5. Particles are compared to merge those that are too close each other, below *τ* centimeters.
6. Then, the system does an extra evaluation of each particle verifying whether they are physically plausible considering the ultrasound distance measurements of adjacent walls (see Figure 7). For each particle of the cloud, its weight is set proportionally to the similarity between the distance calculated in the model according to the user orientation and the ultrasonic distance measured by ultrasonic range sensors using the following equation:
  (1) $$w_{i}=\frac{1}{n}\sum_{k=0}^{n}\frac{\text{min}\left(\delta_{k},\Delta_{k},\Delta_{k}^{\text{max}}\right)}{\text{min}(\text{max}\left(\delta_{k},\Delta_{k}\right),\Delta_{k}^{\text{max}})}\forall i\in \Pi$$
  where *w_i_* is the weight of particle *i* within the cloud Π and n is the number of sensors. *δ_k_*, Δ*_k_* and $\Delta_{k}^{\text{max}}$ are respectively the distance calculated by the system, measured by the range sensor k and the maximum range of the sensor k. If there is no signal for Δ*_k_*, this means that objects are further than $\Delta_{k}^{\text{max}}$, so it is set to $\Delta_{k}^{\text{max}}$ for the weight calculation. Equation 1 measures the similarity between the position estimate and the ultrasonic measurements.
7. Particles with lower weight are then removed from the cloud. The system keeps the fixed maximum of particles *ν* in order to be real time.
8. The system calculates the current user position estimate, displays it on the map of the building, and waits for the next motion event (step 2).

As a result, after each step, the system obtains a set of hypothetical positions, each with a different probability (assigned as a weight). The system then chooses the most plausible user position *p_u_* using a weighted average of the position of all the particles in the cloud using:

(2) $$p_{u}=\frac{\sum_{i=0}^{\nu }w_{i}p_{i}}{\sum_{i=0}^{\nu }w_{i}}$$

where *p_i_* = (*x_i_*, *y_i_*) is the position of particle *i*, and *w_i_* its weight. Once *p_u_* is calculated, it is displayed on the map.

In step 6, the system calculates the distance to the nearest obstacle in the model using the rays sampling approach (see Figure 7). This approach has been chosen for its lower computational needs. The cone shape of the sound wave is split into *λ* rays. The system then calculates the intersection between those rays and the walls represented in the model. The calculation for one ray stops once the ray crosses an obstacle or travels the maximum range of the sensor. For each intersection found, the system will set the wall distance *δ* to the distance between the particle and the intersection of the angle *θ_j_* (radian) between the wall and the ray that satisfies the following equation:

(3) $$\frac{\pi }{2}-\frac{\alpha }{2\lambda }\leq \theta_{j}\leq \frac{\pi }{2}+\frac{\alpha }{2\lambda }$$

where *α* is the aperture angle of the ultrasound sensor. If two wall intersections are calculated for the same particle, the shortest one is kept. This condition allows the system to consider an object as the possible obstacle that reflected the sound.

### 3.3. Components and System Setup

**Figure 8.**
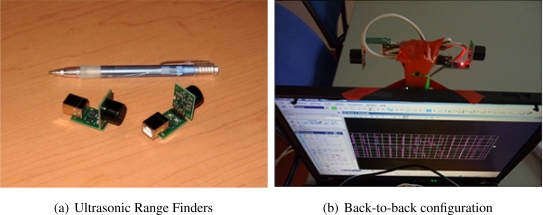
(**a**) The two ultrasound range sensors connected to the USB interface modules. (**b**) The two sensors in a back-to-back configuration installed on top of the portable computer carried by the user.

The prototype uses an IBM ThinkPad laptop equipped with a 2.0 GHz Pentium M processor and 1.5 GB of RAM as well as Microsoft Windows XP and Bentley MicroStation V8. It also uses two Devantech SRF02 Ultrasound Range Finders, each connected to a Devantech USB to I2C Interface Module, enabling us to connect them to our portable computer (see Figure 8(a)). We also used a D-LINK USB hub to connect both sensors to the same USB port on the computer. The total cost of the ultrasound sensor setup with hub and cables was just under $200.

The Xsens MTx three degrees of freedom orientation tracker is fixed on the user’s foot for step and user orientation measurements, and the two ultrasonic range sensors are installed on the back of the computer screen (see Figure 8(b)). The user holds the computer in his hands and keeps it always in the same orientation as his body and his feet (to prevent the ultrasonic range sensor to measure the distance to walls that are not quite adjacent to the user). This could be solved by using an extra orientation sensor attached to the computer, but that was not done in this experiment. The proposed system poses some constraints to the user’s movements. However, those constraints could be alleviated by using a different setup: a smartphone instead of a laptop, ultrasound range sensors installed on the user’s shoulders, extra orientation sensors on the user’s back, *etc.* The simple setup we proposed was aimed at demonstrating that the combination of those technologies could be beneficial.

As the user moves, the user sees his position displayed in real time on a map of the tracking area. The sensors take ranging measurements on both sides. At the speed of a walking human (e.g., 5 km/h), a ranging cycle time of 70 ms means we can get one range measurement every 10 cm along the walls.

The five parameters of the system were given the following values: *ν* = 150, *ε* = 25 cm, *κ* = 20, *τ* = 5 cm and *λ* = 7. The first value was found to be the maximum number for which we could obtain real time measurements on the computer used for the experiments. This is a small number of particles compared with other real time particle based studies [18,19,21]. However, in our tracking system, their is much computation to do for each particles: a lot of processing time is spent calculating, for each particle, the distances to adjacent model elements, and comparing them with the range measurements. The problem is essentially the high number of elements. Solutions to decrease the number of elements and to increase the number of particles are presented in the conclusion. *κ* = 20 was found to be a good value to ensure a uniform distribution within the measurement error *ε* = 25 cm. The value for *ε* has been chosen after many tests showing the maximum drift between two steps was below the selected value. The tests showed good particle distribution with *τ* = 5. *λ* = 7 offered good results considering the computational time required. For the purpose of the project, a fixed step length of 1.25 m has been pre-set in the system; Instead of measuring step length by double integrating acceleration, we set it to a fixed length using a technique described in the following section. The system assumed an error on step length measurements of up to 25 cm.

With this configuration the system can handle one user step per second. If the user walks faster, the system cannot calculate his position fast enough and a delay appears.

### 3.4. Test

**Figure 9.**
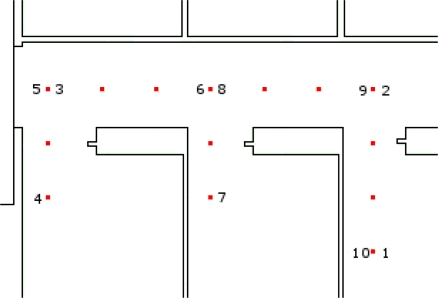
Model of the tracking area. The lines are walls and the squares are floor markers for each step. The numbers refer to the sequence in the test path (1 indicates beginning, and 10, ending of the path).

To test the system, we set up an experiment that was simple to realize and interpret, as a proof of concept for using ultrasound range sensors in human tracking. The test area was on the ground floor of the OTB Building (Delft University of Technology). It was a 2 m wide corridor connected with three rooms of about 3 m × 5 m in size (see Figure 9). The user was asked to walk in such a way to put his feet on each of the floor markers that were fixed on the floor prior to the experiment. The user started from position 1 in Figure 9, and carefully followed the path of floor markers, reaching sequentially positions 2, 3 and so on, until he returned to the starting point (10). The user walked along that path twice in the test. Again, that constraint on user’s movements was set to simplify the system and does not preclude evaluation of the method’s efficiency. We could give more freedom simply by using a more advanced step measurement algorithm.

## 4. Results and Discussion

**Figure 10.**
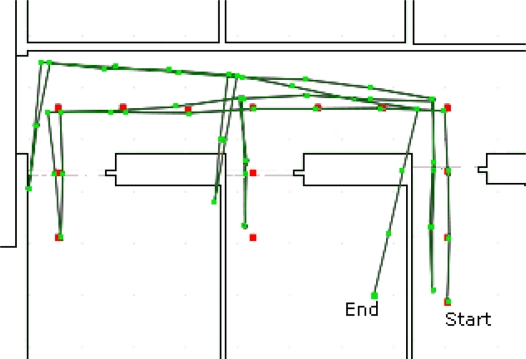
Estimated user position using only the IMU data. The small squares are user positions measured by the system; positions are directly calculated from the data provided by the IMU. The main line represents the link between sequential positions (squares) and are not meant to be interpreted as the estimated user position between those points. The final estimated user position is marked by the *End* marker.

Our first experiment was to estimate the user position using only the IMU, to use as a baseline for comparison. As shown in Figure 10, the first part of the test is quite accurate, but an error on the measurements accumulates and a drifting appears. That is the drift that the proposed system must handle. The positional accuracy progressively decreases and the estimated user position ends up erroneous, inside the wrong room.

**Figure 11.**
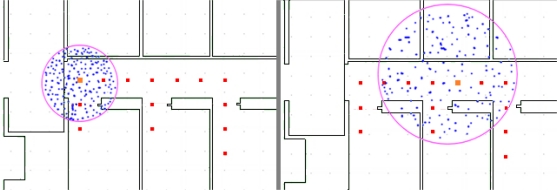
IMU data error representation using the particle filter. Cloud of particles after 9 steps (**left**), 21 steps (**right**). The points represent particles and the circles are the areas covered by the clouds. The circle is a representation of the estimated maximal error of the foot-mounted IMU. The current true user position is marked by the bigger square.

As a first step to compensate for this error, we have added to the system the capacity to estimate the user position using a particle filter. Using the IMU measurements dataset, we observed that the size of the cloud of particles grows quickly from step to step, as a result of the addition of random noise (described in step 3 above), (see Figure 11). With the particle filter, the system knows the area where the user is probably located, but has no clue about his exact location within that area.

**Figure 12.**
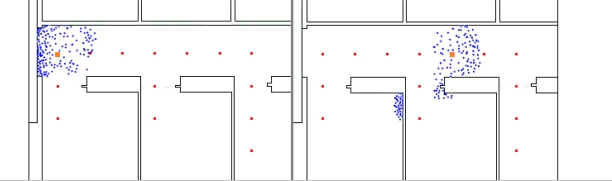
IMU data error representation using the particle filter and the model-based navigation. Cloud of particles after 9 steps (**left**), 21 steps (**right**). The points represent particles. The current true user position is marked by the bigger square. Every particle that crosses a wall has been removed.

The next improvement was adding the model (maps) of the building as a constraint to the displacement of particles. All displacements and positions that are impossible (e.g., particle located inside a wall, or crossing a wall) are eliminated by the system. We applied those constraints to the same dataset. Our results showed that although it helped in providing a better estimate, the resulting accuracy was still insufficient. As shown in Figure 12, the area covered by the cloud of particles is much smaller, resulting in a denser cloud and consequently a better estimation of the user’s position. However, in some cases the cloud can be split.

**Figure 13.**
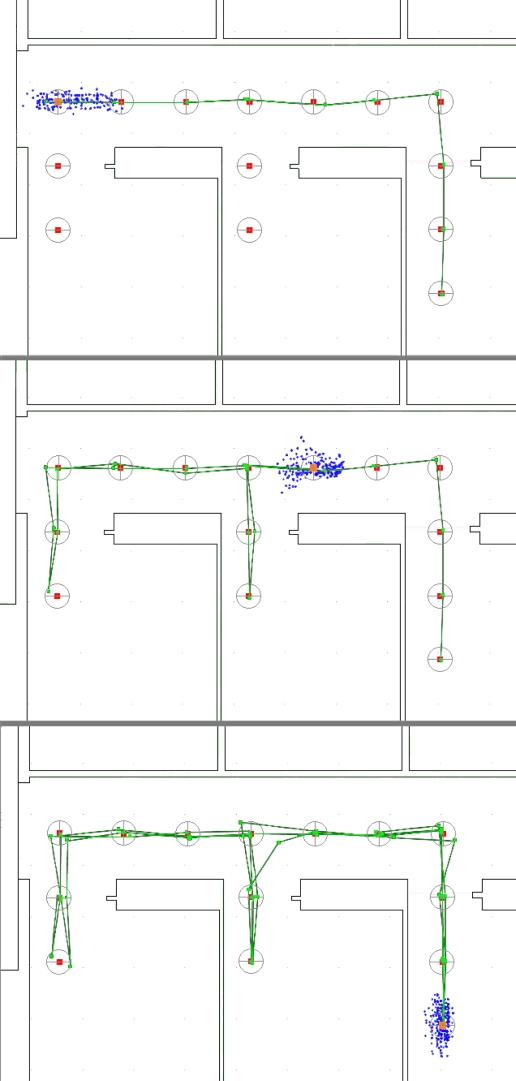
IMU data error representation using the particle filter, the model-based navigation and ultrasonic range measurements. Cloud of particles after 9 steps (**top**), 21 steps (**middle**), 52 steps (**bottom**). The true user position is marked by the center of the circle. The weighted average of the particles positions was chosen as the user position estimate.

Lastly, we added to the system the two ultrasound range sensors. The sensors measured the distance to the walls adjacent to the user and the system eliminated the particles that disagreed with those measurements, therefore eliminating even more hypothetical positions than if using the model alone. Using the same dataset, we observed that the addition of ultrasonic measurements decreased significantly the extent of the cloud of particles (see Figure 13).

In order to operate in real time, the system must limit the number of particles. However, that has the disadvantage of adding a representation error when the cloud becomes too big, as there are not enough particles to ensure a uniform distribution within the cloud. The ultrasonic range measurements provided a way to eliminate hypothetical positions, therefore resulting in a smaller cloud of particles and, as a result, a better accuracy with the same number of particles.

The bottom image in Figure 13 shows the final estimated path for the whole test. The squares along the estimated user path showing the estimated user position for each pass are grouped together, very close to the real user positions. The drift observed in the first experiment has been handled properly, resulting in an estimated position with a very good accuracy.

Table 1 summarizes the position estimation error for the 52 steps test. Max, Min and Ending show respectively the largest, the smallest and the final errors obtained with the tracking system. It shows that the use of ultrasound range sensors clearly increased the system’s accuracy. The experiment was quite simple and straightforward: the model was exact with no other people walking and all ultrasonic measurements perpendicular to the wall. However, because the user’s orientation is obtained from the compass built into the IMU and is not fully reliable (see Figure 10), the system does not receive true user orientation and must calculate wall distances within the model in an angular way, considering the angular response of the ultrasound sensors that allows angular wall distance measurements. The only advantage for the system of the wall perpendicularity is that the ultrasound sensors get accurate measurements at each user’s step.

Finally, there are types of error that cannot currently be handled. The following sources of error are the main limitations of the ultrasonic range approach proposed:

- Discrepancy between the virtual model and the real building.
- Differences between the model and the building caused by furniture (if not available in the model) or passers-by in the tracking area.
- Opened and closed doors.
- Open area, without obstacles within the ultrasonic sensors’ range.

To make the system usable in real world situations, it should be made resilient to a variety of real world environments. In spite of those limitations, we believe the system represents a significant step forward in portable model-based indoor navigation.

## 5. Conclusions

In this study, we developed and tested a fully wearable, real-time personal tracking system, that work in reduced visibility. The system consists of the following components: a foot-mounted IMU, a particle filter, a model-based navigation system and two ultrasound range sensors. The hypothesis that using portable ultrasound range sensors will improve the accuracy of existing indoor localization methods was confirmed. The knowledge about the distance to adjacent walls makes the system capable of compensating for the error that accumulates with IMU measurements. The proposed system exploits the concept of active sensing. It takes advantage of the real time environment configuration, and matches measurements to a virtual model to find the most probable user location. The implementation (algorithms and carefully selected parameters) allow the calculation of user position in real time. We have used simple technologies as proof of concept, but some of the assumptions could be modified to further improve the overall performance of the system.

The system used a constant step length that is not suitable for real situations. The foot-mounted IMU is not reliable for positioning but allows the system to get a coarse estimation of the user’s motion. Moreover, it could provide the confidence that the sensors have in the estimation, based on the calculation of the signal to noise ratio in the signal. That information could be useful to dynamically set the system error radius *ε* and particles’ re-sample value *ν* on the measured motion vectors. It should result in a cloud of particles representing the probability distribution of the user position more accurately.

The speed of model-based navigation could be improved by changing the partitioning of the vector model. Currently we used a grid with a constant size. Woodman *et al.* [6] proposed a polygons representation of the building to speed up the computation and help the localization of the user. An octree-based representation [24] could also increase the computation speed. Alternatively, we could imagine a system that uses a graph or a raster of pre-set positions to estimate the user’s positions. The system could try to figure out which pre-set position has a better correspondence with known information. This offers the possibility of pre-computation of information (e.g., wall distances). The grid should be replaced by a more computationally efficient method to increase the maximum number of particles.

The system has to be tested in more complex environments (e.g., large corridors, rooms with walls that are not parallel, repetitive architectural patterns, *etc.*). As described by Woodman *et al.* [6], the climbing stairs event could also be detected by the IMU. They show that when the user walks up to the next floor, all particles that are not on the same floor as the user can be removed, resulting in a very good error resetting event. In this respect the use of a 3D model (instead of floor plans) could be beneficial.

We could also imagine learning methods (e.g., support vector machine, decision tree) classifying the ultrasonic range measurements in order to take a better decision from that information. Therefore, it might help in situations where the model is not perfectly representative of reality (e.g., in the presence of people, furniture and opened/closed doors).

The current equipment configuration can be enhanced. A smaller computer, (e.g., tablet PC, smartphone) equipped with USB port or Bluetooth could be used instead of a laptop to make the system lighter. The ultrasound sensors could be several (looking ahead and up) and fixed on the user’s clothes (e.g., on the shoulders or near the knees). Each set of ultrasound sensors could move freely with its own compass. Alternatively, sensors could be fixed in order to get measurements at 360° around the user, which could lead to a system that does not need to know the global orientation of ultrasound sensors, but only respective to each other. Eventually, an embedded system could do most of the computation, including a better timing control of the range sensors to better discriminate echoes, leaving the management and the display to a smartphone. A system that would leave the user’s hands free would be ideal.

## Figures and Tables

**Table 1.** Tracking error.

**System:**	**Distance from the true position (m)**				

	*μ*	*σ*	Max	Min	Ending
IMU only	0.59	0.35	1.47	0.01	1.47
with model	0.47	0.30	1.06	0.01	1.06
with ranging	0.13	0.07	0.40	0.03	0.18
